# 2-Hydrazinoquinoline as a Derivatization Agent for LC-MS-Based Metabolomic Investigation of Diabetic Ketoacidosis

**DOI:** 10.3390/metabo3040993

**Published:** 2013-10-31

**Authors:** Yuwei Lu, Dan Yao, Chi Chen

**Affiliations:** Department of Food Science and Nutrition, University of Minnesota, St. Paul, MN 55108, USA; E-Mail: luxxx572@umn.edu (Y.L.); dyao@umn.edu (D.Y.)

**Keywords:** 2-hydrazinoquinoline, LC-MS, chemical derivatization, metabolomics, ketoacidosis, diabetes

## Abstract

Short-chain carboxylic acids, aldehydes and ketones are products and regulators of many important metabolic pathways. Their levels in biofluids and tissues reflect the status of specific metabolic reactions, the homeostasis of the whole metabolic system and the wellbeing of a biological entity. In this study, the use of 2-hydrazinoquinoline (HQ) as a novel derivatization agent was explored and optimized for simultaneous liquid chromatography-mass spectrometry (LC-MS) analysis of carboxylic acids, aldehydes and ketones in biological samples. The formation of carboxylic acid derivative is attributed to the esterification reaction between HQ and a carboxyl group, while the production of aldehyde and ketone derivatives is through the formation of Schiff bases between HQ and a carbonyl group. The compatibility of HQ with biological samples was demonstrated by derivatizing urine, serum and liver extract samples. Using this HQ-based approach, the kinetics of type 1 diabetes-induced metabolic changes was characterized by the LC-MS-based metabolomic analysis of urine samples from streptozotocin (STZ)-treated mice. Subsequently, carboxylic acid, aldehyde and ketone metabolites associated with STZ-elicited disruption of nutrient and energy metabolism were conveniently identified and elucidated. Overall, HQ derivatization of carboxylic acids, aldehydes and ketones could serve as a useful tool for the LC-MS-based metabolomic investigation of endogenous metabolism.

## 1. Introduction

Short-chain carboxylic acids, aldehydes and ketones serve important functions in almost all biological processes inside the body, especially in nutrient and energy metabolism. They are substrates and products of numerous enzymatic reactions, intermediate metabolites in both anabolic and catabolic metabolism, building blocks for complex biomolecules, regulators of metabolic pathways and causes of metabolic disorders and oxidative stress. Analyzing these metabolites in biological samples is essential for understanding the mechanisms behind the homeostasis or disruption of a biological system [1,2,3], but can also be challenging due to various factors, including the interferences of complex biological matrices, inherent low concentrations in samples and the limitation of analytical methods.

Gas chromatography-mass spectrometry (GC-MS) has been a major technical platform for analyzing polar carboxylic acids, aldehydes, ketones and many other endogenous metabolites owing to its chromatographic resolution and sensitive detection, as well as the availability of a comprehensive compound library for structural identification [4]. Since most metabolites in biological samples are nonvolatile or semi-volatile, chemical derivatization is generally required for generating volatile derivatives that are compatible with the GC system. Among many derivatization methods for GC analysis, a tandem oximation-silylation procedure has been widely adopted in practice, in which oximation of aldehydes and ketones is commonly achieved by the reactions between carbonyl group and methoxyamine, while the reactions with silylation agents replace the active hydrogen in alcohols, carboxylic acids, amines and thiols [5,6]. In general, the products of oximation-silylation derivatization are more stable and also have better chromatographic performance than their precursor metabolites [7]. However, to conduct this procedure, water has to be removed from the samples to avoid the degradation of silylation agents, derivatives and GC columns [8]. Because water is the basic solvent of unprocessed biological samples, the derivatization of biological samples is commonly preceded by dehydration processes, such as solvent extraction, evaporation or lyophilization [9,10,11]. All these dehydration processes make the sample preparation in the GC-MS analysis of biological samples time-consuming and inefficient and also could lead to the loss of volatile metabolites, such as acetone, in samples.

Liquid chromatography-mass spectrometry (LC-MS), by its nature, is more compatible with the aqueous matrix of biological samples than GC-MS. However, direct LC-MS analysis of short-chain polar carboxylic acids, ketones and aldehydes without derivatization is still hindered by poor chromatographic performance and low ionization efficiency. Reversed-phase LC columns, as the most commonly used LC columns, are not capable of retaining and separating these hydrophilic metabolites effectively, leading to poor or no signals in the mass detector. The recent development of hydrophilic interaction liquid chromatography (HILIC) has improved the chromatographic separation of polar compounds for MS analysis [12]. However, the instability of reactive carbonyl metabolites, such as acetoacetate and oxaloacetate, in the LC system and the ion source, still prevents reliable detection and measurement of these metabolites [13]. To overcome these challenges, chemical derivatization offers an alternative solution to enhance chromatographic performance, stability and detectability of carboxylic acids, aldehydes and ketones in the LC-MS system [14]. A large selection of LC-MS derivatization agents, such as 2-picolylamine (PA), 2-hydrazinopyridine (HP), 2,4-dinitrophenylhydrazine (DNPH) and dansyl hydrazine (DH), have been developed to react with the carbonyl group in ketones and aldehydes or the carboxyl group in carboxylic acids [15]. However, few of them are capable of effectively reacting with carboxylic acids, aldehydes and ketones all together for simultaneous analysis of these three classes of metabolites.

Diabetes, as the most prevalent metabolic disease in humans, has been widely used as the disease model to examine the technical merit of metabolomics techniques. Nuclear magnetic resonance (NMR), GC-MS and LC-MS-based metabolomics have been used to investigate the metabolic events in diabetes, leading to the observations of diabetes-induced changes in carbohydrate, lipid and amino acid metabolism [16,17,18,19]. However, specific metabolomic investigation of carboxylic acid, aldehyde, and ketone metabolites in diabetes has not been conducted. In this study, 2-hydrazinoquinoline (HQ) was developed as an effective derivatization agent for simultaneous LC-MS analysis of carboxylic acids, aldehydes and ketones in biological samples. The potential use of HQ derivatization to characterize metabolic changes was demonstrated by the LC-MS-based metabolomic analysis of streptozotocin-induced type 1 diabetes.

## 2. Results

### 2.1. Identification of HQ As an Effective Derivatization Agent for LC-MS Detection of Short-Chain Carboxylic Acids, Aldehydes and Ketones

**Figure 1:**
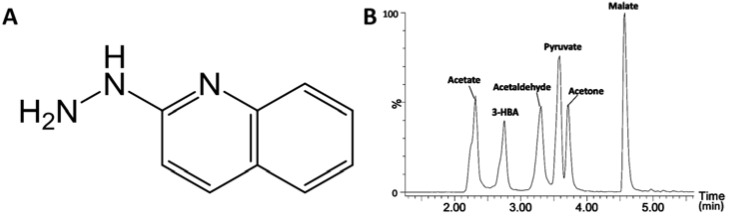
LC-MS analysis of 2-hydrazinoquinoline (HQ) derivatives. (**A**) Structure of HQ; (**B**) a representative chromatogram of HQ derivatives.

In an effort to derivatize short-chain carboxylic acids in urine for LC-MS analysis, we used 2-hydrazinopyridine (HP), an established derivatization reagent [20], to react with carboxylic acids, but observed poor retention of derivatization products in a regular reversed-phase C18 column (data not shown). Subsequently, HQ (Figure 1A), an analog of HP with greater hydrophobicity, was chosen in order to prolong the retention of derivatized carboxylic acids in the reversed-phase LC (RPLC) system. The initial observation from the LC-MS analysis of HQ-derived urine samples indicated that HQ derivatization not only improved the chromatographic performance and ionization efficiency of short-chain carboxylic acid derivatives, but also produced derivatives with various aldehydes and ketones (data not shown). To further confirm this observation and explore the application of HQ in LC-MS-based metabolite profiling and metabolomic analysis, the reactivities of HQ, HP, 2-picolylamine (PA) and dansyl hydrazine (DH) with a mixture of test compounds were compared under a common reaction condition [21]. The selection of PA, HP and DH were based on their known applications as the derivatization agents of carboxylic acids (PA and HP), aldehydes and ketones (DH) [22,23]. Six test compounds, including acetic acid, 3-hydroxybutyrate, malic acid, acetaldehyde, acetone and pyruvic acid, represent monocarboxylic acids, dicarboxylic acids, hydroxyl acids, keto acids, aldehydes and ketones, which are commonly produced in energy and nutrient metabolism. The performances of these derivatization agents were evaluated based on: (1) their capacity to effectively react with six test compounds in the mixture; (2) chromatographic separation of their derivatives by reversed-phase C18 LC column; and (3) the signal intensity of their derivatives in a mass spectrometer. The results showed that HQ derivatization facilitated the separation and detection of all six test compounds under a common condition for LC-MS analysis, including aqueous acetonitrile mobile phases, reverse-phase C18 column and positive electrospray ionization (ESI) mode (Figure 1B and Table 1). In contrast, HP and PA only reacted with carboxylic acids, including acetic acid, 3-hydroxylbutyric acid (HBA), malate and pyruvic acid, while DH did with carbonyl compounds in this mixture, including acetaldehyde, acetone and pyruvic acid (Table 1 and Supplemental Figure 1). Owing to the presence of both carbonyl and carboxylic acid moieties in its structure, pyruvic acid is the only compound that has reacted with all four derivatization agents. Overall, the results clearly indicated that HQ performed better than the other three agents in analyzing six test compounds under defined experimental conditions.

**Table 1:** The derivatization reactions and the molecular formula of derivatization products between derivatization agents (2-picolylamine (PA), 2-hydrazinopyridine (HP), HQ, dansyl hydrazine (DH)) and a mixture of acetic acid, 3-hydroxybutyric acid (HBA), malic acid, acetaldehyde, acetone and pyruvate. The structures and MS/MS spectra of enlisted HQ derivatives are presented in Figure 2. The structures of enlisted PA, HP and DH derivatives are presented in Supplemental Figure 1A–K. N.D. indicates that the derivative is not detected.

Derivatization reactions	Acetate 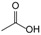	HBA 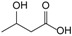	Malate 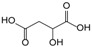	Acetaldehyde 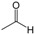	Acetone 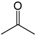	Pyruvate 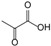
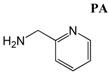	**Forming amide C_8_H_10_N_2_O**	**Forming amide C_10_H_14_N_2_O_2_**	**Forming di-amide C_16_H_18_N_4_O_3_**	**N.D.**	**N.D.**	**Forming amide C_9_H_10_N_2_O_2_**
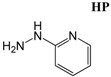	**Forming hydrazide C_7_H_9_N_3_O**	**Forming hydrazide C_9_H_13_N_3_O_2_**	**Forming di-hydrazide C_14_H_16_N_6_O_3_**	**N.D.**	**N.D.**	**Forming hydrazide C_8_H_9_N_3_O_2_**
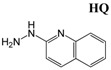	**Forming hydrazide C_11_H_11_N_3_O**	**Forming hydrazide C_13_H_15_N_3_O_2_**	**Forming di-hydrazide C_22_H_20_N_6_O_3_**	**Forming hydrazine C_11_H_11_N_3_**	**Forming hydrazine C_12_H_13_N_3_**	**Forming hydrazine C_12_H_11_N_3_O_2_**
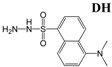	**N.D.**	**N.D.**	**N.D.**	**Forming hydrazine C_14_H_17_N_3_O_2_S**	**Forming hydrazine C_15_H_19_N_3_O_2_S**	**Forming hydrazine C_15_H_17_N_3_O_4_S**

**Figure 2:**
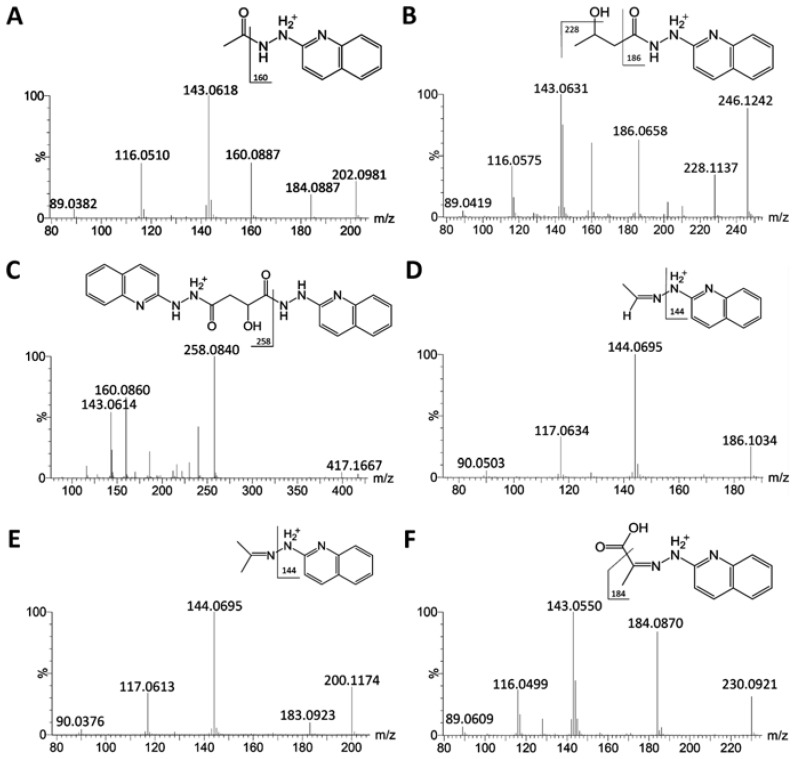
MS/MS spectra of HQ derivatives. (**A**) Acetate-HQ derivative; (**B**) HBA-HQ derivative; (**C**) malate-HQ derivative; (**D**) acetaldehyde-HQ derivative; (**E**) acetone-HQ derivative; (**F**) pyruvate-HQ derivative.

**Scheme 1:**
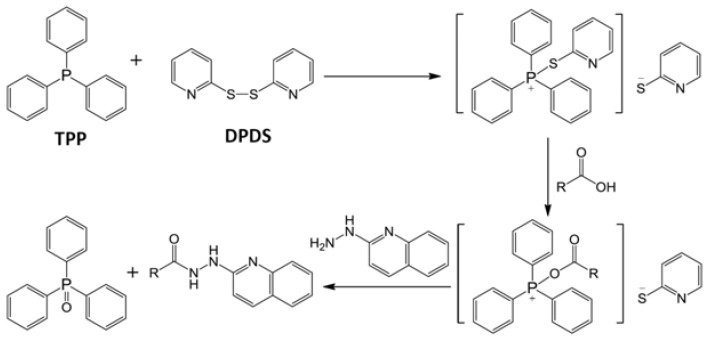
HQ derivatization of carboxylic acids. Carboxylic acids are activated by 2,2′-dipyridyl disulfide (DPDS) and triphenylphosphine (TPP) to form acyloxyphosphonium ions, which then react with HQ to form hydrazides.

**Scheme 2:**
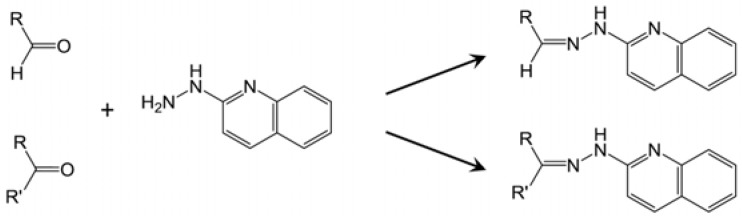
HQ derivatization of aldehydes and ketones. HQ reacts with aldehydes and ketones to form hydrazones.

Analysis of MS/MS fragmentograms of these HQ derivatives (Figure 2A–F) suggested that the derivatization of carboxylic acids was through the formation of hydrazides, while the derivatization of aldehydes and ketones was through the formation of hydrazones. In the reaction mixture, 2,2′-dipyridyl disulfide (DPDS) and triphenylphosphine (TPP) function as the activation agents to convert carboxylic acid to the acyloxyphosphonium ion before it can react with HQ to form a hydrazide bond (Scheme 1) [22]. As for HQ derivatization of aldehydes and ketones, DPDS and TPP are not needed, since the terminal hydrazinyl nitrogen in HQ, a strong nucleophile, directly attacks the carbonyl carbon in aldehydes and ketones, leading to dehydration and the formation of a C=N bond in hydrazone (Scheme 2).

### 2.2. Optimization of HQ-Mediated Derivatization Reaction Conditions

**Figure 3:**
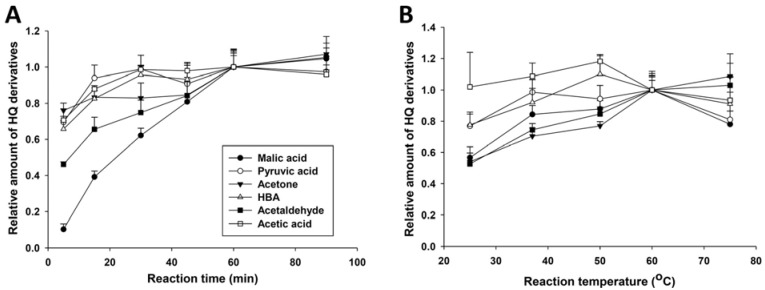
Optimization of HQ derivatization reactions. (**A**). Effect of reaction time on HQ derivatization; (**B**) effect of reaction temperature on HQ derivatization. The amounts of HQ derivatives formed by the incubations at 60 °C for 60 min were arbitrarily set as one. Experimental values are expressed as the mean ± standard deviation (SD).

The influences of solvent, temperature and reaction time on HQ derivatization were examined to optimize the reaction conditions. Acetonitrile was selected as the solvent for derivatization agents and reactions after observing much more effective derivatization reactions in acetonitrile than the ones in methanol, ethanol and water (data not shown). The optimal reaction time was determined by monitoring the kinetics of HQ derivatization reactions. Among six test compounds, the derivatization of acetic acid, HBA, acetone and pyruvate was almost complete within the first 15 min of the reactions, while the derivatization of malic acid and acetaldehyde continued within the first 60 min (Figure 3A). Therefore, 60 min was selected as the reaction time for the following HQ derivatization reactions. Furthermore, 60 °C was chosen as the optimal reaction temperature for six test compounds after comparing the reaction rates at 25, 37, 50, 60 and 75 °C (Figure 3B).

### 2.3. Compatibility of HQ Derivatization with Biological Samples

**Figure 4:**
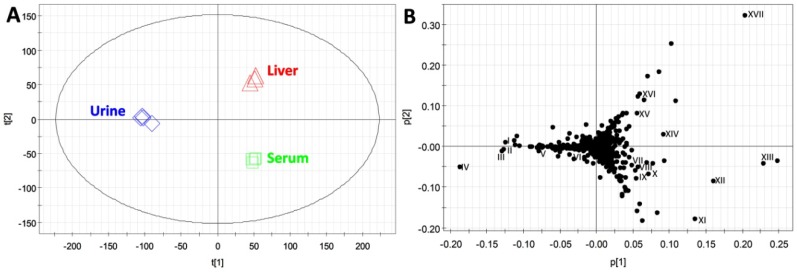
LC-MS-based metabolomic analysis of HQ-derivatized serum, urine and liver extract samples from wild-type mice. (**A**) The scores plot of a principal components analysis (PCA) model on three groups of biological samples (n = 4). The *t*[1] and *t*[2] values represent the scores of each sample in the principal components 1 and 2, respectively. (**B**) The loadings plot of ions detected by LC-MS analysis. The *p*[1] and *p*[2] values represent the contributing weights of each ion to the principal components 1 and 2 of the PCA model, respectively. Major contributing ions in each sample group are labeled (I–XVII).

Results from the proof-of-concept experiments revealed that HQ can derivatize a mixture of carboxylic acids, aldehydes and ketones dissolved in water (Figure 1, Figure 2 and Figure 3). To further test whether HQ could function as an effective derivatization agent for simultaneous detection of carboxylic acids, aldehydes and ketones in biological samples, urine, serum and liver extract samples from wild-type mice were derivatized by HQ and then analyzed by LC-MS-based metabolomics. Comparable signals from spiked deuterated acetic acid (internal standard) were observed after LC-MS analysis of HQ-derivatized urine, serum and liver samples, indicating that different matrices of these biological samples did not significantly affect HQ derivatization. Through the principal components analysis (PCA) of LC-MS data, HQ-derivatized urine, serum and liver extract samples were distinctively separated in a two-component model (Figure 4A). HQ derivatives contributing to the classification of these biological samples in the PCA model were further identified in a loadings plot (Figure 4B).

Subsequent structural analyses confirmed the presence of different carboxylic acids, aldehydes and ketones in urine, serum and liver and further revealed the capacity of HQ to derivatize structurally diverse metabolites (Table 2). For example, a group of amino acid metabolites, including α-ketoisovaleric acid (I), formiminoglutamic acid (II), α-ketoisocaproic acid (III), α-ketoglutaric acid (IV) and 4-hydroxyphenylacetic acid (VI), are abundant in the urine samples, while the metabolites important in energy metabolism, such as pyruvic acid (VII), acetic acid (VIII), glucose (IX), HBA (XI), lactic acid (XII) and acetone (XIII), are well correlated with the serum samples (Figure 4B and Table 2). Interestingly, formaldehyde (XIV), propionic acid (XV), dehydroascorbic acid (XVI) and acetaldehyde (XVII) are identified as the abundant metabolites in the liver, even though these metabolites have not been commonly reported in previous studies on hepatic metabolome (Figure 4B and Table 2). Since urine, serum and liver are widely used for examining the metabolic status of human and animals, the result from this metabolomic analysis suggested that HQ derivatization is well suitable for analyzing carboxylic acids, aldehydes and ketones in biological samples.

**Table 2:** A list of confirmed HQ derivatives of carboxylic acid, aldehyde and ketone metabolites. Information on each metabolite includes its molecular formula, the formula of its HQ derivative and the exact mass of the protonated HQ derivative ([M+H]^+^). The metabolites contributing to the separation of urine, serum and liver extracts in Figure 4A were presented with their identities (ID) labeled in Figure 4B. The chemical structures of these intermediary metabolites were confirmed by comparing their chromatographic peaks and MS/MS fragmentograms with the standards. U, S and L indicate the significant presence of specific metabolites in urine, serum and liver, respectively, based on the signals detected by the mass spectrometer.

Compounds	ID	Formula	Derivative Formula (No. of HQ moiety)	Exact mass of [M+H]^+^	Distribution
Formaldehyde	XIV	CH_2_O	C_10_H_9_N_3_ (1)	172.0875	L
Acetaldehyde	XVII	C_2_H_4_O	C_11_H_11_N_3_ (1)	186.1031	L
Acetic acid	VIII	C_2_H_4_O_2_	C_11_H_11_N_3_O (1)	202.0980	S
Acetone	XIII	C_3_H_6_O	C_12_H_13_N_3_ (1)	200.1188	U/S/L
Propionic acid	XV	C_3_H_6_O_2_	C_12_H_13_N_3_O (1)	216.1137	U/L
Pyruvic acid	VII	C_3_H_4_O_3_	C_12_H_11_N_3_O_2_ (1)	230.0930	S/L
Lactic acid	XII	C_3_H_6_O_3_	C_12_H_13_N_3_O_2_ (1)	232.1086	S/L
Acetoin	V	C_4_H_8_O_2_	C_13_H_15_N_3_O (1)	230.1288	U
Butyric acid	-	C_4_H_8_O_2_	C_13_H_15_N_3_O (1)	230.1288	-
Acetoacetic acid	-	C_4_H_6_O_3_	C_13_H_13_N_3_O_2_ (1)	244.1086	U
HBA	XI	C_4_H_8_O_3_	C_13_H_15_N_3_O_2_ (1)	246.1243	S
Fumaric acid	-	C_4_H_4_O_4_	C_13_H_11_N_3_O_3_ (1)	258.0873	
Succinic acid	-	C_4_H_6_O_4_	C_13_H_13_N_3_O_3_ (1)	260.1030	U
Malic acid	-	C_4_H_6_O_5_	C_22_H_20_N_6_O_3_ (2)	417.1675	-
α-Ketoisovaleric acid	I	C_5_H_8_O_3_	C_14_H_15_N_3_O_2_ (1)	258.1243	U
α-Ketoglutaric acid	IV	C_5_H_6_O_5_	C_14_H_13_N_3_O_4_ (1)	288.0984	U
Dehydroascorbic acid	XVI	C_6_H_6_O_6_	C_15_H_13_N_3_O_5_ (1)	316.0928	U/L
α-Ketoisocaproic acid	III	C_6_H_10_O_3_	C_15_H_17_N_3_O_2_ (1)	272.1399	U
Glucose	IX	C_6_H_12_O_6_	C_15_H_19_N_3_O_5_ (1)	322.1397	S/L
Mannose	X	C_6_H_12_O_6_	C_15_H_19_N_3_O_5_ (1)	322.1397	S/L
Citric acid	-	C_6_H_8_O_7_	C_13_H_11_N_3_O_3_ (1)	258.0873	U
Formiminoglutamic acid	II	C_6_H_10_N_2_O_4_	C_15_H_17_N_5_O_3_ (1)	316.1404	U
4-Hydroxyphenylacetic acid	VI	C_8_H_8_O_3_	C_17_H_15_N_3_O_2_ (1)	294.1237	U
4-Hydroxyphenylpyruvic acid	-	C_9_H_8_O_4_	C_18_H_15_N_3_O_3_ (1)	322.1192	U

### 2.4. Application of HQ Derivatization in LC-MS-based Metabolomic Analysis of Diabetes-Induced Ketoacidosis

**Figure 5:**
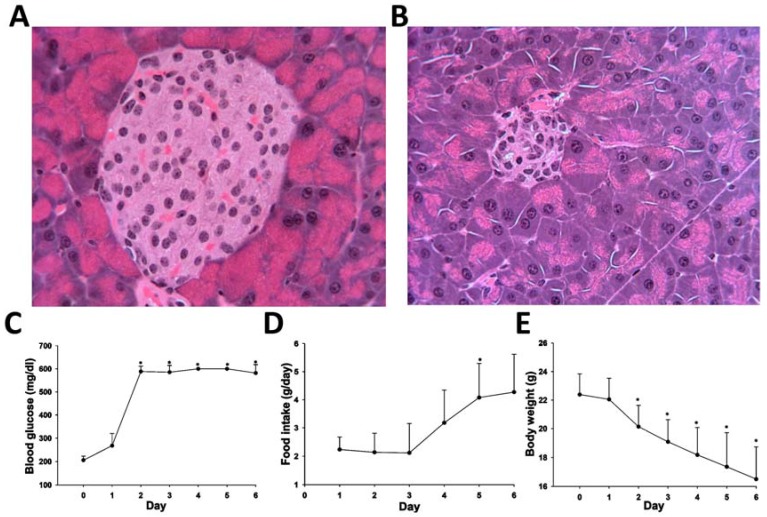
Phenotypes of streptozotocin (STZ)-induced Type 1 diabetes mouse model. (**A**) Histology of pancreatic islets in control mice; (**B**) histology of pancreatic islets six days after STZ treatment; (**C**) blood glucose concentration from day 0 to day 6 of STZ treatment (the upper limit of quantification of the glucose meter is 600 mg/dL); (**D**) food intake from day 1 to day 6 after STZ treatment; (**E**) body weight from day 0 to day 6 of STZ treatment. * indicates *p* < 0.05.

Considering ketoacidosis is the most prominent metabolic phenotype of type 1 diabetes, the newly-established HQ derivatization method was adopted to examine the diabetes-induced metabolic changes in mice. Type 1 diabetes was achieved by injecting a single high dose of streptozotocin (STZ) (180 mg/kg) to the mice. The influences of STZ treatment on pancreas were examined by histological analysis. Hematoxylin and eosin (H&E) staining showed that compared to the control mice, the number and the size of β-cell islets in pancreas were greatly reduced in the STZ-treated mice (Figure 5A,B). Consistent with these histological changes, hyperglycemia developed rapidly after STZ treatment and was sustained during six days of sample collection (Figure 5C). Body weight of STZ-treated mice decreased dramatically (Figure 5E), even though food intake increased significantly two days after STZ treatment (Figure 5D). In addition, water consumption and urine volume also increased dramatically after STZ injection (data not shown). All these results confirmed that STZ-treated mice exhibited typical phenotypes of type 1 diabetes.

**Figure 6:**
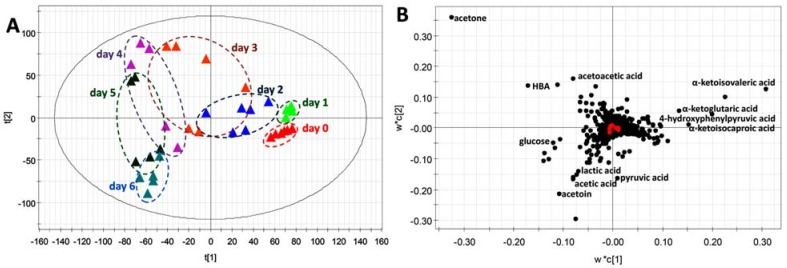
LC-MS-based metabolomic analysis of STZ-induced metabolic changes in mice. Urine samples were derivatized by HQ before LC-MS analysis. (**A**) The score plot of a PLS-DA model on urine samples from control and STZ-treated mice. The *t*[1] and *t*[2] values represent the scores of each sample in the principal components 1 and 2, respectively. (**B**) The loading plot of urinary ions contributing to the time-dependent separation of HQ-derivatized urine samples in the PLS-DA model. The w*c[1] and w*c[2] values represent the contributing weights of each ion to the principal components 1 and 2 of the PLS-DA model, respectively. Major urinary ions affected by STZ treatment were labeled with their chemical identities.

The influences of STZ treatment on the urinary metabolome were examined by HQ derivatization and LC-MS analysis of urine samples collected before and after STZ treatment. After the data were processed by supervised partial least squares-discriminant analysis (PLS-DA), time-dependent separation of urine samples was observed in a two-component model (Figure 6A), indicating that a combination of HQ derivatization and LC-MS analysis is capable of revealing the kinetics of STZ-induced metabolic changes in type 1 diabetic mice. Subsequently, the metabolites contributing to the separation of sample groups were identified in the loading plot of the PLS-DA model, and the chemical identities of these HQ derivatives were determined (Figure 6B).

**Figure 7:**
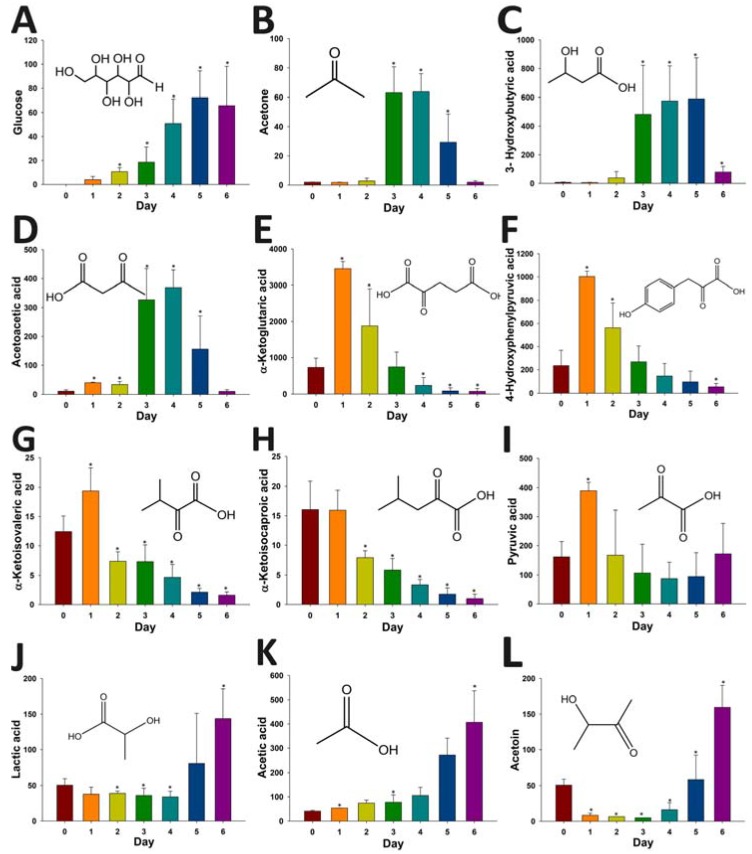
Influences of STZ treatment on carboxylic acid, aldehyde and ketone metabolites in mouse urine. The relative abundances of urinary metabolites identified in Figure 6B were determined by calculating the ratio between the signal ion counts (SIC) of the metabolite of interest and the total ion counts (TIC) of a sample. * indicates *p* < 0.05. (**A**) glucose; (**B**) acetone; (**C**) HBA; (**D**) acetoacetic acid; (**E**) α-ketoglutaric acid; (**F**) 4-hydroxyphenylpyruvic acid; (**G**) α-ketoisovaleric acid; (**H**) α-ketoisocaproic acid; (**I**) pyruvic acid; (**J**) lactic acid; (**K**) acetic acid; (**L**) acetoin.

Several interesting patterns of metabolite distribution were observed after analyzing the relative abundances of these metabolites in pre-and post-STZ urine samples (Figure 7). For example, the levels of glucose (an aldehyde) and ketone bodies (acetone, HBA and acetoacetate) increased after STZ treatment (Figure 7A–D). However, the patterns of these changes in urinary glucose and ketone bodies were different. Glucose was absent in pre-STZ urine, and its level steadily increased after STZ treatment (Figure 7A). In contrast, the elevation of ketone bodies appeared to be transient as their levels peaked on day 3 and 4, but decreased afterwards (Figure 7B–D). Another group of urinary metabolites that were greatly affected by STZ treatment were α-keto acids (Figure 7E–H). The levels of α-ketoglutaric acid and 4-hydroxyphenylpyruvic acid, which are α-keto acid metabolites of glutamate and tyrosine, increased at first after STZ treatment, but decreased thereafter and became even lower than the controls (Figure 7E,F). In addition, two branched-chain amino acid metabolites, α-ketoisovaleric acid from valine and α-ketoisocaproic acid from leucine, also decreased dramatically by STZ treatment, even though a transient increase of α-ketoisovaleric acid was observed on day 1 (Figure 7G,H). Pyruvate, lactate and acetate are three important intermediates in intermediary metabolism. Pyruvate level was largely unaffected after STZ treatment, except a transient increase on day 1 (Figure 7I). Lactate, as the product of nicotinamide adenine dinucleotide (NADH)-dependent reduction of pyruvate, remained unchanged in the first four days of STZ treatment, but its level increased on day 5 and 6 (Figure 7J). The profile of acetic acid was similar to the profile of glucose, as its level in urine gradually increased after the treatment (Figure 7K). Acetoin (3-hydroxy-2-butanone) was another ketone metabolite affected by STZ. Interestingly, its level in urine decreased dramatically after STZ treatment, but started to increase on day 4 and rose to a much higher level than the controls (Figure 7L). Overall, the observed metabolic changes in carboxylic acid, aldehyde and ketone metabolites are largely consistent with the expected metabolic phenotypes of type 1 diabetics, suggesting that HQ derivatization facilitated the LC-MS-based metabolomic analysis of diabetic ketoacidosis.

## 3. Discussion

As essential intermediates and end products of nutrient and energy metabolism, short-chain carboxylic acids, aldehydes and ketones in tissues and biofluids are sensitive to metabolic disturbance and, thus, can function as the biomarkers of metabolic events caused by exogenous challenges and disease development. Targeted analyses of these metabolite biomarkers are commonly used in clinical diagnosis of inherited or acquired metabolic diseases [24]. To meet the needs of targeted analysis, the majority of chemical derivatization agents for LC-MS analysis only aim at reacting with one functional group in the molecules of interest, such as the carbonyl group in ketones and aldehydes or the carboxyl group in carboxylic acids, since the specific reactivity of these derivatization agents is well suited for targeted analysis of individual metabolites. However, for comprehensive metabolomic analysis of a biological system or exploratory investigation of a metabolic disorder, the derivatization agents that can facilitate simultaneous measurement of diverse metabolites are advantageous. In this study, HQ was identified as an effective derivatization agent for LC-MS analysis of carboxylic acids, aldehydes and ketones in biological samples (Figure 1, Figure 2, Figure 3 and Figure 4). Furthermore, the utilization of HQ derivatization in LC-MS-based metabolomic analysis of STZ-treated mice not only confirmed the expected ketoacidotic phenotype of type 1 diabetes, but also revealed the kinetics of STZ-induced metabolic changes and potential correlations among these changes (Figure 5, Figure 6 and Figure 7). Compared to the results from LC-MS-based metabolomic analysis of underivatized urine samples from the same experiment (data not shown), these results are more relevant to the disruption of carbohydrate, fatty acid and amino acid metabolism in type 1 diabetes. Considering that the metabolites detected by HQ-based LC-MS analysis are important intermediates or end products in nutrient and energy metabolism, the HQ derivatization method has the potential to find more applications in examining the homeostasis and disorder of the metabolic system.

The conditions of HQ derivatization have been examined in this study. Aldehydes and ketones directly react with the hydrazine group in HQ to form hydrazones, while TPP and DPDS-mediated activation of carboxylic acid is required for the formation of hydrazides with HQ (Scheme 1 and Scheme 2). Compared to the nitrogen atom in the amine group of PA, the terminal nitrogen in the hydrazine group of HQ is much more nucleophilic for the reaction with positively-charged carbon in the carbonyl group of aldehydes and ketones (Table 1). However, the versatility of HQ in the derivatization of carboxylic acids, aldehydes and ketones cannot be solely attributed to its hydrazine moiety, since HP and DH also contain a hydrazine moiety in their structures. Therefore, besides hydrazine, other structural features of HQ, HP and DH also contribute to their reactivity (Table 1). Compared to the pyridine moiety of HP, the quinoline moiety of HQ could function as a better electron donor for the nucleophilic activity of the hydrazine group, which may explain why HQ can react with ketones and aldehydes, but not HP. As for the differences between DH and HQ, two possible mechanisms might contribute to the incapability of DH to derivatize carboxylic acids: (1) the sulfonyl group in DH can withdraw electrons from its hydrazine group, making it less reactive for reactions with carboxylic acid; (2) water in the six-compound test solution and biological samples may negatively affect the reactions between DH and carboxylic acid. In fact, the formation of DH derivatives of carboxylic acids (acetic acid and propionic acid) was observed in a preliminary experiment when the reactions were conducted in an anhydrous condition (data not shown). The exact mechanism behind this observation is not determined, but could be contributed by the altered reactivity of DH in the presence of water, as reported previously [25]. In contrast, HQ derivatization reactions can proceed with about 5% water content in the reaction mixture, suggesting that HQ derivatization is compatible with the water-based biological samples. It should be noted that the lower limit of detection of HQ-based metabolite analysis was not thoroughly evaluated here, since the main goal of developing HQ as a derivatization agent is to expand the coverage, instead of the sensitivity, of metabolite detection in metabolomic analysis. In addition, for quantitative analysis, the full scan method (50–1,000 m/z) in the quadrupole time-of-flight (QTOF) mass spectrometer used in this study is not as sensitive as the multiple reaction monitoring (MRM) method used in triple quadrupole mass spectrometers. However, based on the signals of identified metabolites and their chemical standards in the mass spectrometers, the concentrations of carboxylic acid, aldehyde and ketone metabolites in the urine and serum samples of control and diabetic mice range from a few micromolar to a few millimolar. Targeted metabolite analysis in the future will provide more detailed information on the sensitivity of the HQ derivatization method.

Diabetic ketoacidosis in type 1 diabetes is triggered by insulin deficiency and subsequent excess of counteractive hormones (glucagon, catecholamines and cortisol). Marked hyperglycemia, ketosis and acidosis have been defined as three elements of diabetic ketoacidosis [26]. The observation of increased glucose, ketone bodies and carboxylic acids (lactate and acetate) in STZ-treated mice confirmed these metabolic phenotypes of diabetic ketoacidosis (Figure 7). In addition, this observation also correlated with the decrease of multiple α-keto acids from amino acid metabolism (Figure 7) since ketogenic and glucogenic amino acids are major sources of glucose and ketone bodies in diabetes [27]. The decreases of 4-hydroxyphenylpyruvic acid, α-ketoisovaleric acid and α-ketoisocaproic acid were likely caused by the enhanced degradation of tyrosine, leucine and valine, since the upregulation of α-keto acid dehydrogenases that are responsible for their degradation have been observed in diabetic animal models [27,28,29]. α-Ketoglutaric acid is a key intermediate in the tricarboxylic acid (TCA) cycle. The observed decrease in its level reflects the cataplerotic effects of type 1 diabetes [30]. To the best of our knowledge, the changes in urinary acetoin observed in this study have not been reported previously, even though it is known that acetoin can be formed in bacteria, animals and humans through the metabolism of acetolactate, pyruvate and ethanol [31,32]. Further studies are required to understand the metabolic events behind the diabetes-induced changes in acetoin, as well as the significance of these changes.

## 4. Experimental Section

### 4.1. Reagents

HQ, 3-hydroxylbutyric acid (HBA), butyric acid, fumaric acid, propionic acid, mannose, acetoin, α-ketoglutaric acid and triphenylphosphine (TPP) were purchased from Alfa Aesar (Ward Hill, MA, USA). Acetone, acetaldehyde, 2-picolylamine (PA), glucose, acetic acid, succinic acid, formaldehyde, 4-hydroxyphenylpyruvic acid and deuterated acetic acid (^2^H_4_-acetic acid) were purchased from Sigma-Aldrich (St. Louis, MO, USA). 2,2′-Dipyridyl disulfide (DPDS), dansyl hydrazine (DH), pyruvic acid, acetoacetic acid and lactic acid were purchased from MP Biomedicals (Santa Ana, CA, USA). Streptozotocin (STZ) was purchased from Calbiochem (San Diego, CA, USA). 2-Hydrazinopyridine (HP), malic acid, 4-hydroxyphenylacetic acid, sodium citrate, LC-MS-grade water and acetonitrile were purchased from Fisher Scientific (Houston, TX, USA).

### 4.2. Animal Treatment and Sample Collection

Male C57BL/6 mice (10 to 12-week-old) from Charles River Laboratories (Wilmington, MA, USA) were used in this study. All animals were maintained in a University of Minnesota (UMN) animal facility under a standard 12 h light/12 h dark cycle with food and water *ad libitum*. Handling and treatment procedures were in accordance with animal study protocols approved by the UMN Institutional Animal Care and Use Committee. STZ treatment was performed following the protocol recommended by the Animal Models of Diabetic Complications Consortium [33]. Prior to STZ treatment, mice were fasted for 4 h. Freshly-prepared STZ solution (buffered by sodium citrate, pH 4.5) was administered by a single intraperitoneal injection to mice at the dose of 180 mg/kg. Drinking water containing 1% sucrose was supplied overnight after the injection to prevent the mortality caused by STZ-induced acute hypoglycemia. Serum samples were collected by submandibular bleeding. Urine samples were collected by housing mice in the metabolic cages for 24 h. Liver and other tissue samples were harvested after animals were euthanized by carbon dioxide. All urine, serum and tissue samples were stored at −80 °C before further analysis.

### 4.3. Biochemical and Histological Analysis of STZ-Induced Diabetes

The glucose concentration in tail vein blood was monitored using an Omnis blood glucose meter (Miramar, FL, USA). Hyperglycemia was defined by a blood glucose level that is higher than 250 mg/dL [34]. The pancreas tissue was immediately fixed in 10% formalin solution after dissection and then embedded in paraffin, sectioned and stained with hematoxylin and eosin (H&E) for general histology.

### 4.4. Preparation of Aqueous Liver Extraction

The aqueous fraction of liver tissue was prepared using Bligh’s method [35]. Liver tissue was homogenized in methanol. After adding chloroform and water, the extraction solution was vortexed and then centrifuged at 18,000 × *g* for 10 min to separate lipid and aqueous phases. The aqueous phase of the liver extract was transferred to a 1.5-mL tube and stored at −80 °C before further analysis.

### 4.5. Derivatization of Six Test Compounds with PA, HP, DH and HQ

The test solution contained 500 µM acetic acid, 50 µM HBA, 2 mM malic acid, 200 µM acetaldehyde, 1 mM acetone and 400 µM pyruvic acid in water. Acetonitrile was used as the solvent for the stock solutions of PA, HP, HQ, DH, DPDS and TPP. To compare the performance of four derivatization agents, 5 µL of test solution was added into a 100 µL of freshly-prepared acetonitrile solution containing 1 mM DPDS, 1 mM TPP and 1 mM derivatization agent (PA, HP, HQ or DH). The reaction mixture was incubated at 60 °C for 15 min, chilled on ice and then mixed with 100 µL of H_2_O. After centrifugation at 18,000 × *g* for 10 min, the supernatant was transferred into a HPLC vial for LC-MS analysis.

### 4.6. Optimization of HQ Derivatization Reactions

To determine the kinetics of HQ derivatization reactions, 5 µL of six-compound test solution was added into 100 µL of freshly-prepared acetonitrile solution containing 1 mM DPDS, 1 mM TPP and 1 mM HQ. The reaction mixture was incubated at 60 °C for 5, 15, 30, 45, 60 or 75 min. The optimal reaction temperature was defined by conducting the same HQ derivatization reactions at 25, 37, 50, 60 or 75 °C for 60 min. The HQ derivatives in the reaction mixture were analyzed by LC-MS.

### 4.7. HQ Derivatization of Biological Samples

Urine, serum or liver extract was spiked with 200 µM ^2^H_4_-acetic acid as the internal standard. The HQ derivatization reaction was conducted by mixing 5 µL of biological sample with 100 µL of acetonitrile solution containing 1 mM DPDS, 1 mM TPP and 1 mM HQ. The reaction mixture was incubated at 60 °C for 60 min. The HQ derivatives in the reaction mixture were analyzed by LC-MS.

### 4.8. LC-MS Analysis of Derivatization Products

The processed reaction mixture from chemical derivatization of standards or biological samples was injected into an Acquity^TM^ ultra-performance liquid chromatography (UPLC) system (Waters, Milford, MA, USA) and separated in an Acquity^TM^ UPLC C18 column (2.1 × 50 mm) by a gradient of the mobile phase ranging from 0.05% aqueous acetic acid containing 2 mM ammonium acetate to 95% aqueous acetonitrile containing 0.05% acetic acid and 2 mM ammonium acetate over a 10-min run. LC elute was introduced into a Waters SYNAPT^TM^ QTOF mass spectrometer (QTOF-MS) (Waters, Milford, MA, USA) for MS analysis. Capillary voltage and cone voltage were maintained at 3.2 kV and 30 V, respectively, in positive electrospray ionization (ESI) mode. Source temperature and desolvation temperature were set at 120 °C and 350 °C, respectively. Nitrogen was used as both the cone gas (50 L/h) and desolvation gas (700 L/h) and argon as the collision gas. For accurate mass measurement, the mass spectrometer was calibrated with sodium formate solution (range *m/z* 50–1,000) and monitored by the intermittent injection of the lock mass leucine enkephalin ([M+H]^+^ = 556.2771 *m/z*) in real time. Mass chromatograms and mass spectral data were acquired and processed by MassLynx^TM^ software (Waters, Milford, MA, USA) in centroid format. Additional structural information was obtained by tandem MS (MS/MS) fragmentation with collision energies ranging from 15 to 40 eV.

### 4.9. Chemometric Analysis of LC-MS Data

Chromatographic and spectral data from HQ-derivatized biological samples were deconvoluted by MarkerLynx^TM^ software. A multivariate data matrix containing information on sample identity, ion identity (retention time and *m/z*) and ion abundance was generated through centroiding, deisotoping, filtering, peak recognition and integration. To avoid the influences of unreacted derivatization agents on chemometric analysis, the ions from these chemicals were excluded from the data matrix. The intensity of each ion was calculated by normalizing the single ion counts (SIC) *vs.* the total ion counts (TIC) in the whole chromatogram. The data matrix was further exported into SIMCA-P+^TM^ software (Umetrics, Kinnelon, NJ, USA) and transformed by mean-centering and Pareto scaling, a technique that increases the importance of low abundance ions without significant amplification of noise. Principal components analysis (PCA) or partial least squares-discriminant analysis (PLS-DA) was adopted to model the data from LC-MS analysis of HQ-derivatized biological samples [36]. Major latent variables in the data matrix were described in a score scatter plot of a multivariate model. Metabolites were identified by analyzing ions contributing to the principal components and to the separation of sample groups in the loading scatter plot. The chemical identities of the metabolites of interest were determined by accurate mass measurement, elemental composition analysis, MS/MS fragmentation and comparisons with authentic standards, if available.

### 4.10. Statistics

Experimental values are expressed as the mean ± standard deviation (SD). Statistical analysis was performed with the two-tailed Student’s *t-*tests for unpaired data, with a *p-*value of <0.05 considered statistically significant.

## 5. Conclusions

In summary, HQ was identified as an effective derivatization agent for simultaneous LC-MS analysis of carboxylic acids, aldehydes and ketones. The capacity of HQ derivatization to characterize the metabolic changes in biological samples was demonstrated by the LC-MS-based metabolomic analysis of type 1 diabetic mice. Because of its simple procedure and its compatibility with water-based samples, HQ derivatization has the potential to become a useful tool for LC-MS-based comprehensive metabolomic profiling, complementary to the existing metabolomic methods for lipids, amino acids and other classes of metabolites.

## Supplementary Materials

Supplementary materials can be accessed at: http://www.mdpi.com/2218-1989/3/4/993.

Supplementary File 1 Supplementary Materials (PDF, 122 KB)

## References

[1] Kumps A., Duez P., Mardens Y. (2002). Metabolic, nutritional, iatrogenic, and artifactual sources of urinary organic acids: A comprehensive table. Clin. Chem..

[2] Laffel L. (1999). Ketone bodies: A review of physiology, pathophysiology and application of monitoring to diabetes. Diabetes Metab. Res. Rev..

[3] Draper H.H., Csallany A.S., Hadley M. (2000). Urinary aldehydes as indicators of lipid peroxidation *in vivo*. Free Radical Bio. Med..

[4] Jellum E., Stokke O., Eldjarn L. (1973). Application of gas chromatography, mass spectrometry, and computer methods in clinical biochemistry. Anal. Chem..

[5] Knapp D.R. (1979). Handbook of Analytical Derivatization Reactions.

[6] Hong Z., Lin Z., Liu Y., Tan G., Lou Z., Zhu Z., Chai Y., Fan G., Zhang J., Zhang L. (2012). Innovative microwave-assisted oximation and silylation procedures for metabolomic analysis of plasma samples using gas chromatography-mass spectrometry. J. Chromatogr. A.

[7] Pierce A.E. (1968). Silylation of Organic Compounds: a Technique for Gas-Phase Analysis.

[8] Grob K., Li Z.W. (1989). Introduction of water and water-containing solvent mixtures in capillary gas-chromatography: 1. Failure to produce water-wettable precolumns (retention gaps). J. Chromatogr. A.

[9] Kind T., Tolstikov V., Fiehn O., Weiss R.H. (2007). A comprehensive urinary metabolomic approach for identifying kidney cancer. Anal. Biochem..

[10] Lanz C., Patterson A.D., Slavik J., Krausz K.W., Ledermann M., Gonzalez F.J., Idle J.R. (2009). Radiation metabolomics. 3. Biomarker discovery in the urine of gamma-irradiated rats using a simplified metabolomics protocol of gas chromatography-mass spectrometry combined with random forests machine learning algorithm. Radiat. Res..

[11] Zhang Q., Wang G., Du Y., Zhu L., Jiye A. (2007). GC/MS analysis of the rat urine for metabonomic research. J. Chromatogr. B.

[12] Jian W., Edom R.W., Xu Y., Weng N. (2010). Recent advances in application of hydrophilic interaction chromatography for quantitative bioanalysis. J. Sep. Sci..

[13] Bajad S.U., Lu W., Kimball E.H., Yuan J., Peterson C., Rabinowitz J.D. (2006). Separation and quantitation of water soluble cellular metabolites by hydrophilic interaction chromatography-tandem mass spectrometry. J. Chromatogr. A.

[14] Xu F., Zou L., Liu Y., Zhang Z., Ong C.N. (2011). Enhancement of the capabilities of liquid chromatography-mass spectrometry with derivatization: General principles and applications. Mass Spectrom. Rev..

[15] Santa T. (2011). Derivatization reagents in liquid chromatography/electrospray ionization tandem mass spectrometry. Biomed. Chromatogr..

[16] Higashi T., Ichikawa T., Inagaki S., Min J.Z., Fukushima T., Toyo’oka T. (2010). Simple and practical derivatization procedure for enhanced detection of carboxylic acids in liquid chromatography-electrospray ionization-tandem mass spectrometry. J. Pharmaceut. Biomed..

[17] Zhao L., Liu X., Xie L., Gao H., Lin D. (2010). 1H NMR-based metabonomic analysis of metabolic changes in streptozotocin-induced diabetic rats. Anal. Sci..

[18] Li X., Xu Z., Lu X., Yang X., Yin P., Kong H., Yu Y., Xu G. (2009). Comprehensive two-dimensional gas chromatography/time-of-flight mass spectrometry for metabonomics: Biomarker discovery for diabetes mellitus. Anal. Chim. Acta.

[19] Newgard C.B., An J., Bain J.R., Muehlbauer M.J., Stevens R.D., Lien L.F., Haqq A.M., Shah S.H., Arlotto M., Slentz C.A. (2009). A branched-chain amino acid-related metabolic signature that differentiates obese and lean humans and contributes to insulin resistance. Cell Metab..

[20] Huffman K.M., Shah S.H., Stevens R.D., Bain J.R., Muehlbauer M., Slentz C.A., Tanner C.J., Kuchibhatla M., Houmard J.A., Newgard C.B. (2009). Relationships between circulating metabolic intermediates and insulin action in overweight to obese, inactive men and women. Diabetes Care.

[21] Maestri L., Ghittori S., Imbriani M., Capodaglio E. (1994). Determination of 2,5-hexandione by high-performance liquid chromatography after derivatization with dansylhydrazine. J. Chromatogr. B.

[22] Matsueda R., Maruyama H., Ueki M., Mukaiyama T. (1971). Peptide synthesis by oxidation-reduction condensation. Ii. The use of disulfide as an oxidant. Bull. Chem. Soc. Jpn..

[23] Tanaka K., Hine D.G., West-Dull A., Lynn T.B. (1980). Gas-chromatographic method of analysis for urinary organic acids. I. Retention indices of 155 metabolically important compounds. Clin. Chem..

[24] Binding N., Klaning H., Karst U., Potter W., Czeschinski P.A., Witting U. (1998). Analytical reliability of carbonyl compound determination using 1,5-dansylhydrazine-derivatization. Fresen. J. Anal. Chem..

[25] Kitabchi A.E., Umpierrez G.E., Murphy M.B., Barrett E.J., Kreisberg R.A., Malone J.I., Wall B.M. (2003). Hyperglycemic crises in patients with diabetes mellitus. Diabetes Care.

[26] Felig P., Wahren J., Sherwin R., Palaiologos G. (1977). Amino acid and protein metabolism in diabetes mellitus. Arch. Intern. Med..

[27] Felig P., Marliss E., Ohman J.L., Cahill C.F. (1970). Plasma amino acid levels in diabetic ketoacidosis. Diabetes.

[28] Aftring R.P., Miller W.J., Buse M.G. (1988). Effects of diabetes and starvation on skeletal muscle branched-chain alpha-keto acid dehydrogenase activity. Am. J. Physiol..

[29] Stanley J.C., Fisher M.J., Pogson C.I. (1985). The metabolism of l-phenylalanine and l-tyrosine by liver cells isolated from adrenalectomized rats and from streptozotocin-diabetic rats. Biochem. J..

[30] Owen O.E., Kalhan S.C., Hanson R.W. (2002). The key role of anaplerosis and cataplerosis for citric acid cycle function. J. Biol. Chem..

[31] Hassinen I. (1963). Acetoin as a metabolite of ethanol. Acta Physiol. Scand..

[32] Casazza J.P., Song B.J., Veech R.L. (1990). Short chain diol metabolism in human disease states. Trends Biochem. Sci..

[33] Tesch G.H., Allen T.J. (2007). Rodent models of streptozotocin-induced diabetic nephropathy. Nephrology.

[34] Clee S.M., Attie A.D. (2007). The genetic landscape of type 2 diabetes in mice. Endocr. Rev..

[35] Bligh E.G., Dyer W.J. (1959). A rapid method of total lipid extraction and purification. Can. J. Biochem. Physiol..

[36] Chen C., Kim S. (2013). LC-MS-based metabolomics of xenobiotic-induced toxicities. Comput. Struct. Biotechnol. J..

