# Supplementary material for: 2-Hydrazinoquinoline as a Derivatization Agent for LC-MS-Based Metabolomic Investigation of Diabetic Ketoacidosis
Source: Metabolites. 2013 Oct 31;3(4):993–1010. doi: 10.3390/metabo3040993 (PMC3937830; doi:10.3390/metabo3040993)

Supplementary Materials

Supplemental Figure 1. PA, HP and DH derivatives of carboxylic acids, aldehydes and ketones. Molecular formula and chemical structures of these derivatives were determined by accurate mass measurement, elemental composition analysis and MS/MS fragmentation. The value of $[M+H]^+$ is the mass-to-charge ratio (*m/z*) of the protonated derivative. (**A**) Acetate-PA; (**B**) HBA-PA; (**C**) malate-PA; (**D**) pyruvate-PA; (**E**) acetate-HP; (**F**) HBA-HP; (**G**) malate-HP; (**H**) pyruvate-HP; (**I**) acetaldehyde-DH; (**J**) acetone-DH; (**K**) pyruvate-DH.

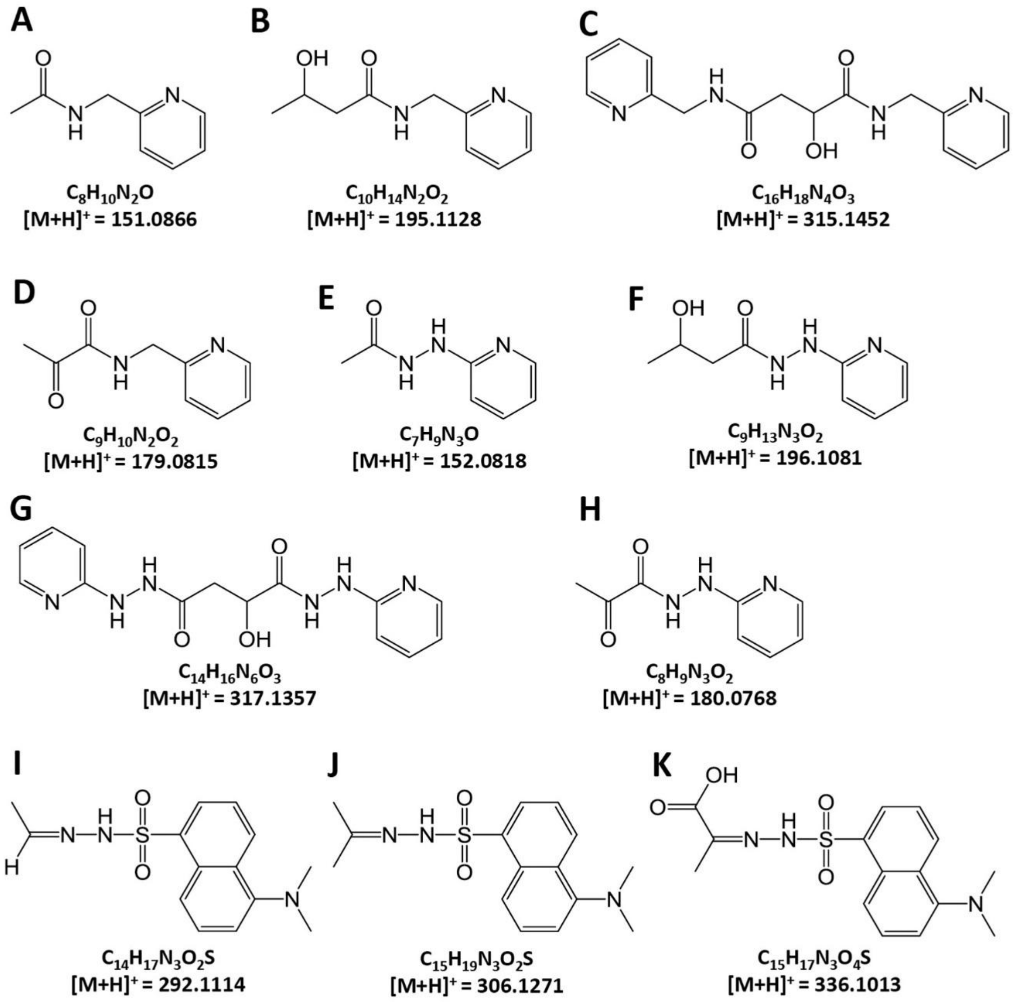

Supplement: Supplementary File 1 — Supplementary Materials (PDF, 122 KB) [file metabolites-03-00993-s002.pdf]
